# Evaluation of fluoroscopic-guided lumbar medial branch block: A cadaveric study

**DOI:** 10.1016/j.inpm.2026.100762

**Published:** 2026-04-20

**Authors:** John Tran, Elysia Chau, Melissa Calleja, Eldon Loh

**Affiliations:** a Division of Anatomy, Department of Surgery, University of Toronto, Toronto, Canada; b Department of Physical Medicine and Rehabilitation, Western University, London, Canada; c Parkwood Institute Research, Lawson Research Institute, London, Canada

**Keywords:** Anatomy, Lumbar, Fluoroscopy, Diagnostic block

## Abstract

**Introduction:**

The gold standard for selecting patients for lumbar radiofrequency ablation (RFA) is medial branch block (MBB). An ideal lumbar MBB would anesthetize the targeted medial branch, with minimal to no spread to surrounding structures. Injectate spread can be affected by numerous factors, including injection volume, and location. Optimizing these factors is important to improve specificity and minimize the number of false positive responders. Capture of unintended structures is relevant as it may impact specificity of the MBB and warrants further investigation. Therefore, the objective of the current study was to perform fluoroscopic guided lumbar MBBs in cadaveric specimens and conduct meticulous dissection to assess dye spread and nerve capture rates.

**Materials and methods:**

Fluoroscopy-guided contrast and dye injection (n = 20) was performed targeting lumbar MB (L1-L5). Following dye injection (0.25 mL), each specimen was meticulously dissected to expose the course of the lumbar dorsal rami branches relative to dye spread. Photographs were taken to document the extent of dye spread and structures that were stained. The capture rate was quantified and reported as a percentage.

**Results:**

A total of 20 fluoroscopy-guided lumbar MBB were performed. Location of contrast flow on fluoroscopy was consistent with the location of dye spread observed in dissected specimens. Dissection post injection consistently found injectate staining all branches of the lumbar dorsal ramus. Injectate was found to spread anteriorly to the lateral aspect of the vertebral body.

**Conclusion:**

Fluoroscopy guided lumbar MBB using contrast and dye were performed in anatomical specimens to assess spread and nerve capture rates. Lumbar MBB at small volumes stained all branches of the lumbar dorsal ramus suggesting it is non-selective. Further anatomical and clinical research is required.

## 1 Introduction

Lumbar medial branch (MB) radiofrequency ablation (RFA) is commonly performed to treat facetogenic low back pain [[1], [2], [3]]. However, the success of lumbar MB RFA can be inconsistent [[2], [3], [4]]; one previous study reported only 39% of patients achieved ≥50% pain relief at the 6-month timepoint [5]. Poor patient selection is an important factor that may contribute to lower success rates following lumbar RFA. The gold standard for selecting patients for lumbar RFA is medial branch block (MBB), which involves the injection of a small amount of local anesthetic over the relevant medial branches supplying a particular lumbar facet. An ideal lumbar MBB would anesthetize the targeted medial branch, with minimal to no spread of injectate to surrounding structures. Injectate spread can be affected by numerous factors, including injection volume, and location of injection. Optimizing these factors is important to improve lumbar MBB specificity, and to minimize the number of false positive responders.

In the previous anatomical literature, only one study was found that investigated lumbar MBB injectate spread [6]. Using fluoroscopic guidance, with subsequent CT imaging and dissection, Wahezi et al. compared dye spread using injectate volumes of 0.25 ml and 0.50 ml in 6 cadaveric specimens. Based on their findings, it was recommended that 0.25 ml was more appropriate to diagnose facetogenic low back pain. However, Wahezi et al. did not quantify nor report nerve capture of other structures, such as the lateral and intermediate branches of the lumbar dorsal ramus. Capture of unintended structures is relevant as it may impact specificity of the MBB to select appropriate patients for lumbar MB RFA and warrants further investigation. Therefore, the objective of the current study was to perform fluoroscopic guided lumbar MBBs in cadaveric specimens and conduct meticulous dissection to assess dye spread and nerve capture rates.

## 2 Material and methods

This anatomical study was conducted in the Division of Anatomy at the University of Toronto. Cadaveric specimens used in this study were procured from the Willed Body Program, and the study was approved by the University of Toronto Health Sciences Research Ethics Board protocol #27210.

### 2.1 Fluoroscopy-guided diagnostic medial branch block

Fluoroscopy-guided dye injection (n = 20) was performed targeting lumbar MB (L1-L5) in 2 light embalmed cadaveric specimens bilaterally. The injection technique/landmarking was performed based on previously published literature [7]. The vertebral endplate at the targeted level was first squared off. The fluoroscope was then brought into an oblique position to optimize the “scotty dog” view, with a sharp delineation of the pedicle. A needle was inserted on the oblique view to the pedicle, ensuring that the needle was above the mamillo-accessory notch, and lateral to the mamillary process. An AP and lateral view were taken to ensure appropriate placement along the expected course of the MB. Specifically, the needle tip was at the superior border of the pedicle isthmus and below the junction of the superior articular and transverse processes. To simulate real world protocol, the contrast solution (0.25 ml) was first injected, with repeat oblique, AP and lateral views captured to document contrast spread on these views. Dye (0.25 mL) was then injected and repeat oblique, AP and lateral views were captured. Fluoroscopic images of the contrast and contrast plus dye injections were documented for subsequent analysis.

### 2.2 Cadaveric dissection and nerve capture

Following the injection, each specimen was meticulously dissected to expose the first (L1) to fifth (L5) lumbar dorsal rami. First, the skin and superficial fascia was removed to expose the erector spinae muscle group. Meticulous dissection and removal of muscle fiber bundles was performed to isolate the lateral and intermediate branches of the lumbar dorsal rami as it courses through the muscles as in situ. The L1-L5 lateral/intermediate branches were traced proximally to their origin off the dorsal ramus and extent of dye spread was documented. Next, careful removal of muscles and fat tissue was performed to expose the course of the MB relative to dye spread. Anterior dissection to assess and document the dye spread was also performed by removing abdominal/pelvic visceral and muscles. Photographs were taken to document the extent of dye spread and structures that were stained. The capture of the lateral, intermediate, and medial branches of the lumbar dorsal rami was determined if the nerve was stained with dye. The capture rate was quantified and reported as a percentage.

## 3 Results

**Fig. 1:**
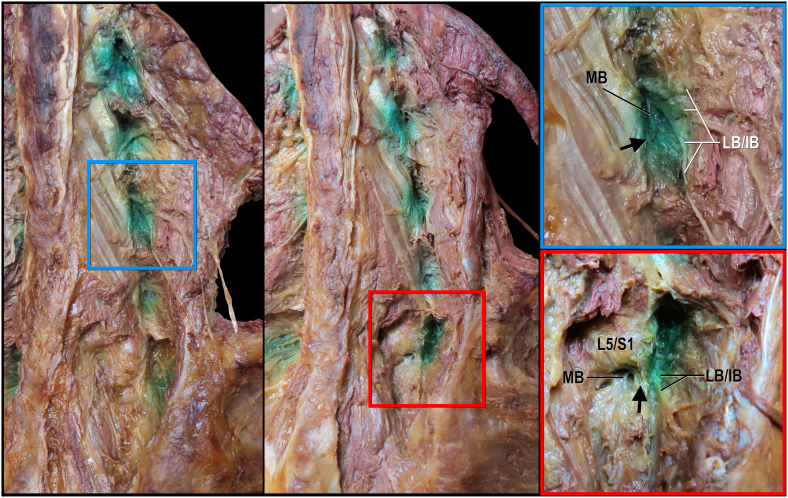
**Cadaveric dissection of branches of the L1-L5 lumbar dorsal rami following contrast and subsequent dye injection, oblique views.** Black arrow indicates mammillo-accessory ligament; IB, intermediate branches; LB, lateral branches; MB, medial branch.

A total of 20 fluoroscopy-guided lumbar MBB were performed. All 20 lumbar dorsal rami (L1-L5) with their branches were dissected. Spread of injected dye was exposed through dissection and documented (Fig. 1) at 19 of 20 levels. In one injection targeting the L3 MB, dye was missing due to the accidental oversight of injecting only contrast; this level was excluded from further analysis. In all specimens, the MB was found along the lateral neck of the superior articular process (SAP), while the other branches of the dorsal ramus (lateral and intermediate) coursed posterolaterally through muscle tissue.

**Fig. 2:**
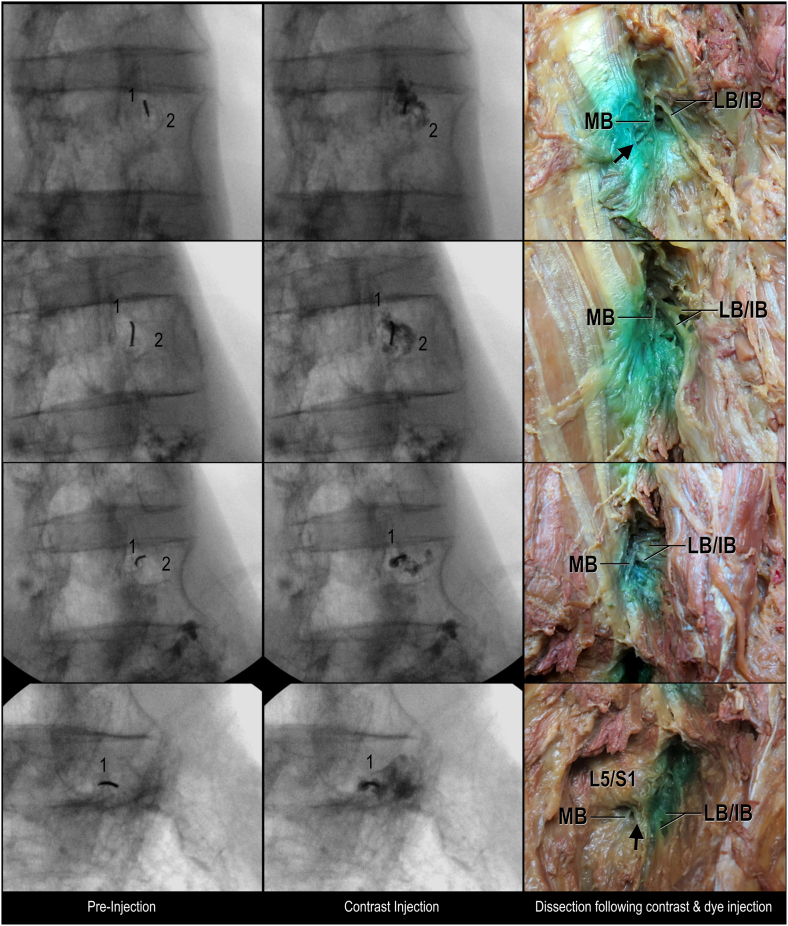
**Fluoroscopy correlation with dissected specimens showing L2-L5 dorsal rami, oblique views.** Black arrow indicates mammillo-accessory ligament; IB, intermediate branches; LB, lateral branches; MB, medial branch; 1, superior articular process/mamillary process; 2, transverse process.

**Supplementary Figure 1:**
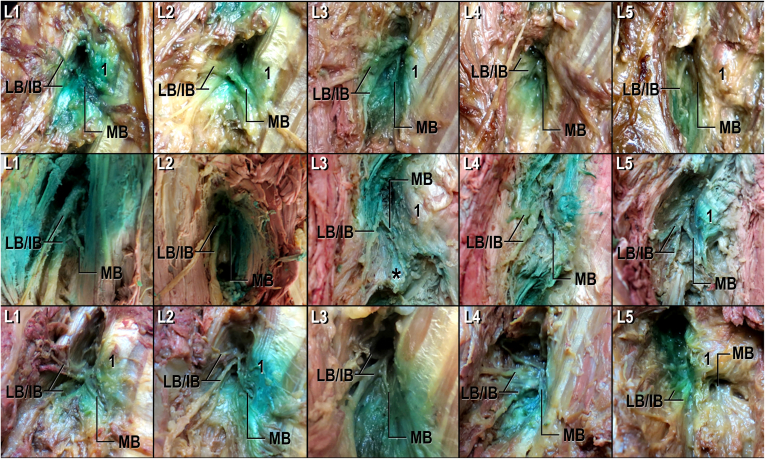
Collection of dissection images showing the branches of the first to fifth lumbar dorsal rami stained with dye. Asterisk, accessory process; MB, medial branch; LB/IB, lateral and intermediate branches; 1, mammillary process.

**Supplementary Figure 2:**
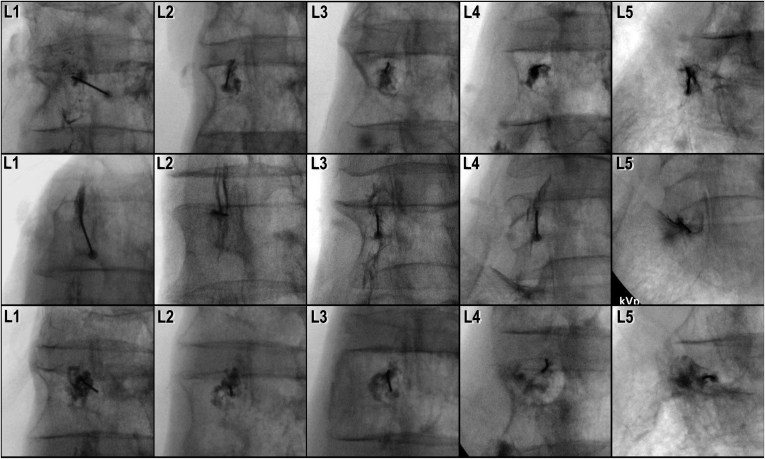
Collection of fluoroscopic images showing contrast flow patterns following medial branch block injections in cadaveric specimens.

In all 19 injections analyzed, there was contrast and dye spread along the junction between the lateral neck of the SAP and transverse process (Fig. 2 and Supplementary Fig. 1). However, there was also contrast and dye spread laterally, encompassing the lateral/intermediate branches of lumbar dorsal ramus in 18 out of 19 specimens (Fig. 2 and Supplementary Fig. 1). Location of contrast flow on fluoroscopy was consistent with the location of dye spread on dissection; that is, dye was located in the same areas as the observed contrast flow (Fig. 2 and Supplementary Figs. 1 and 2).

**Fig. 3:**
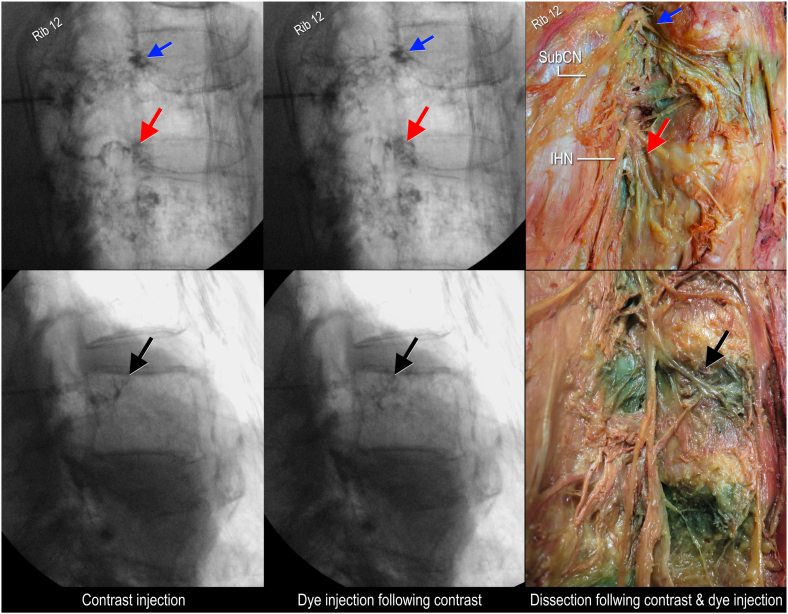
**Anterior spread of contrast with dye as seen on fluoroscopy images and dissected specimens, lateral views.** Blue arrow indicates contrast around the anterior ramus; Red arrow, contrast and dye around the intervertebral foramen; Black arrow, pooling of contrast and dye along the lateral aspect of the vertebral body staining rami communicantes; IHN, iliohypogastic nerve; SubCN, subcostal nerve. (For interpretation of the references to colour in this figure legend, the reader is referred to the Web version of this article.)

Anterior spread through the intervertebral foramen was observed in 16 out of 19 injections (Fig. 3), with staining of the anterior surface of the transverse process. In 12 out of these 16 injections, contrast injection and dye were observed in proximity to the sympathetic chain, and as far anterior as the lateral aspect of the vertebral body, deep to psoas major (Fig. 3 & Supplementary video 1). The majority of injections with anterior spread to the lateral vertebral body was found in one specimen (10 out of 10). In the second specimen, only 2 out of 10 injections had anterior spread as far as the lateral vertebral body (Supplementary video 2). Supplementary video related to this article can be found at https://doi.org/10.1016/j.inpm.2026.100762

The following are the supplementary data related to this article:

Multimedia component 1 Multimedia component 1

Multimedia component 2 Multimedia component 2

Frequency of nerve capture of the different branches of the lumbar dorsal ramus was consistent. In 18 out of 19 injections (94.7%) included in the analysis, dye was found to stain all branches of the lumbar dorsal ramus.

## 4 Discussion

Positive response to diagnostic lumbar MBB is the current standard to select patients for RFA. Consequently, the ability of the block to capture only the MB is a critical criterion. However, the spread pattern and nerve capture rate of this standard diagnostic procedure has not been thoroughly investigated. This study documents two important findings that have potentially important clinical implications. First, lumbar MBBs in cadaveric models showed non-selective capture of branches of the lumbar dorsal ramus. Second, there is anterior spread capturing nerves supplying anterior structures, including the intervertebral discs. These two findings were present despite the use of small volumes (0.25 mL of contrast followed by 0.25 mL of dye), highlighting the challenge of minimizing false positive rates following lumbar MBBs.

In this study, injection resulted in high capture rate of the MB as well as the lateral and intermediate branches of the L1 to L5 dorsal rami (18 out of 19 specimens). The only prior anatomical study examining injectate spread during MBB was published by Wahezi et al., who reported that “medial branches at the injection sites were coated in all specimens”; no comment was made regarding capture of other branches of the lumbar dorsal ramus. The findings of the current study provide anatomical evidence that the MBB can capture not only the MB, but other branches of the lumbar dorsal ramus. This suggests that current injection protocols are non-specific for the MB. It also provides anatomical evidence that MBBs may potentially alleviate low back pain from other structures. For example, since the superior cluneal nerves have been reported to originate from the T12-L5 lateral branches of the dorsal rami [8,9], it is conceivable that blockade of these branches during diagnostic MBBs may temporarily alleviate chronic low back pain due to undiagnosed cluneal neuralgia [[10], [11], [12], [13], [14]].

A number of case reports have been published describing successful treatment of cluneal neuralgia alleviating chronic low back pain [[15], [16], [17]], with the prevalence of superior and middle cluneal nerve neuralgia estimated to be as high as 14% and 13%, respectively [10,15]. In a recent scoping review, it was concluded that “… superior and middle cluneal nerve entrapment is under-recognized and therefore undertreated. This lesion should be included as part of the differential diagnosis of low back pain … ’ [18]. The non-selective capture of medial, lateral and intermediate branches of the dorsal rami with MBB may contribute to false positive responses, leading to failed lumbar MB RFA despite positive diagnostic MBB.

In addition to diffuse capture of the medial, lateral and intermediate branches of the dorsal rami in the current cadaveric study, dissection and radiographic evidence showed that dye and contrast flowed anteriorly through the intervertebral foramen to the posterolateral aspect of the vertebral body. Although there may be differences between cadaveric specimens and live patients that contribute to discrepancies in dye spread along tissue planes, the reported anterior spread is consistent with the findings of a previous imaging study [19].Dreyfuss et al. identified contrast spread to the epidural space and intervertebral foramina in 16% of injections on CT scan following MBB in healthy volunteers, with contrast volumes of 0.5 mL [19]. While anterior spread of contrast during MBB may conceivably provide pain relief in those with discogenic or radicular involvement, a prior study demonstrated that false positive MBB (with 0.5 mL local anesthetic) was not more common in those with discogenic and/or radicular pain. In fact, those without discogenic pain (as identified on discography) had a higher percentage of false positive MBB [20]. It is possible that other structures innervated by small sensory nerves in the foramen, or from the sympathetic chain, may be responsible for false positive MBBs, regardless of discogenic/radicular involvement.

The non-selective nature of lumbar MBB is clinically relevant and highlights the importance of understanding and interpreting contrast flow patterns when diagnostic blocks are used. Based on contrast flow analysis and understanding of the relevant anatomy, clinicians should consider other potential pain generators beyond the lumbar facet joints when there is no response to RFA despite positive MBB. Additional targeted blocks in these cases (e.g., cluneal diagnostic block) may be indicated. Further anatomical and clinical research is required to validate this postulation.

One major limitation of the current study is the small sample size (20 injections in 2 cadaveric specimen) which may not document all the variations of nerve course and injectate flow patterns making the findings non-generalizable to the broader population. Additionally, cadaveric specimens may not perfectly mimic physiological conditions in living tissue; therefore, dye spread in cadaveric specimens may differ from those in vivo. Consequently, there may be exaggerated spread of injectate in anatomical studies. Previous histological studies, however, have reported that the tissue quality of lightly embalmed specimens are comparable to living tissue [21,22]. Other potential causes for anterior spread include variations in patient-specific anatomy and/or needle placement techniques. The possibility of anterior spread is of particular importance in the context of chemical neurolysis, which is growing in interest as a potential treatment modality for joint pain [23,24]. Clinicians should be mindful of potential anterior spread patterns and the possibility of unintentional damage to anterior structures. Future larger studies should be conducted to validate the current findings including the need for in vivo imaging studies to assess contrast spread. Lastly, the use of dye staining as a surrogate of nerve capture is limited as dye staining may not directly correlate with nerve block. That is, the presence of staining may not necessarily result in significant anesthesia. However, the findings of this study suggest there is a possibility that these structures may be blocked during diagnostic MBB. A future contrast flow imaging analysis and correlation with sensory deficit may help further validate this study's findings. Regardless of the sample size, physiological condition, and presence of staining, the finding of injectate spreading diffusely even at small volumes (0.25 mL of contrast followed by 0.25 mL of dye) highlights the ongoing challenge of minimizing false positive results following MBB and selecting the best candidates for RFA.

## 5 Conclusions

In this cadaveric study, fluoroscopy guided lumbar MBB using contrast and dye were performed to assess the spread and nerve capture rates. Using meticulous dissection, lumbar MBB at small volumes were found to capture all branches of the dorsal ramus suggesting it is non-selective. Moreover, injectate was found to spread through the intervertebral foramen as far anterior as the lateral aspect of the vertebral body potentially capturing sensory afferents supplying other pain generators in the lumbar spine. Further anatomical and clinical research is required.

## Conflict of interest

JT & EL have received research grants from International Pain and Spine Intervention Society (IPSIS), Avanos Medical Inc., and FUSmobile Inc. (paid directly to Lawson Research Institute). JT is a consultant with Brixton Biosciences Inc. and Merz Therapeutics (relationship ended). EL is a consultant with Brixton Biosciences Inc. The other authors have nothing to disclose.
